# Diatomite-like KFeS_2_ for Use in High-Performance Electrodes for Energy Storage and Oxygen Evolution

**DOI:** 10.3390/nano13040643

**Published:** 2023-02-06

**Authors:** Can Wang, Kailin Li, Qing Sun, Shijin Zhu, Chenzhi Zhang, Yunhao Zhang, Zhongyi Shi, Youzhong Hu, Yuxin Zhang

**Affiliations:** 1 College of Materials Science and Engineering, Chongqing University, Chongqing 400044, China; 2 Multi-Scale Porous Materials Center, School of Chemistry and Chemical Engineering, Chongqing University, Chongqing 400044, China; 3 School of Energy and Power Engineering, Chongqing University, Chongqing 400044, China; 4 Undergraduate School, Chongqing University, Chongqing 400044, China

**Keywords:** KFeS_2_, diatomite, hydrothermal synthesis, supercapacitors, oxygen evolution reaction

## Abstract

Bifunctional materials possess remarkable properties that allow them to store and convert electrical energy easily. In this paper, diatomite-like potassium iron disulfide (KFeS_2_) was synthesized by a multistep sacrificial template method, and its morphological, electrochemical, and oxygen evolution reaction (OER) properties were investigated. KFeS_2_ was found to be porous, hollow, and cake-like, which suggests a high specific surface area (SSA) and abundant electrochemically active sites. A very high specific capacitance of 651 F g^−1^ at 1.0 A g^−1^ was also obtained due to the substance’s unique structure and high porosity. Additionally, the diatomite-like KFeS_2_ possessed a very low overpotential ƞ_10_ of 254 mV at a current density of 10 mA cm^−2^ and a small Tafel slope of about 48.4 mV dec^−1^. Thus, the diatomite-like KFeS_2_ demonstrates broad application prospects for both energy storage and conversion.

## 1. Introduction

Energy storage and conversion are two key points related to many proposed solutions for serious environmental problems to energy production, and both have already received considerable academic attention over the years [1,2,3,4]. As a type of electrochemical energy storage device, supercapacitors can be widely used in many “new energy” vehicles, camera flashes, and energy back-up systems because of their higher power density, faster charge and discharge rates, and longer cycle lives compared to conventional batteries [5,6,7]. In addition, the electrochemical energy conversion pathway of the oxygen evolution reaction (OER), which is one half of the electrochemical decomposition of water, is environmentally friendly, low cost, emits no carbon, and can be used to produce clean hydrogen energy. OER has also already been widely studied, and the performance of supercapacitance and OER relies on the active materials used. Iron-based compounds have been used as a primary electrode material for supercapacitors due to the various valence states of Fe, as well as their high specific capacitance, wide potential window, low cost, and their own environmental friendliness [8,9]. Among them, iron-based sulfides have become one of the most important electrode materials for supercapacitors due to their high electrical conductivity and unique physicochemical properties [10,11,12,13]. Furthermore, iron-based sulfide nanomaterials are considered to be efficient electrocatalytic materials due to their high abundance, low toxicity, and high electrochemical activity [14,15,16,17,18].

Among all iron-based compounds, ternary KFeS_2_ possesses variable physical and chemical properties because of its mixed-valence Fe [19,20]. Thus, ternary KFeS_2_ shows broad application prospects in energy storage and electrocatalysis [21,22,23,24,25,26]. However, the electrochemical properties of most KFeS_2_ nanostructures are much lower than their theoretical values because of their low specific surface area (SSA) resulting in poor electrochemically active sites [27,28,29]. Additionally, KFeS_2_ has also been found to exhibit the phenomenon of easy agglomeration [21]. Therefore, shaping KFeS_2_ into a unique morphology that both prevents agglomeration and enhances its SSA would have immediate benefits.

To this end, as a sacrificial template material, diatomite has become one of the most popular templates for preparing porous structures due to its own high porosity, low volumetric weight, high chemical stability, and high SSA [30,31]. In addition, the three-dimensional structure of diatomite has been shown to be able to solve the problem of material agglomeration effectively [6,32,33]. Thus, in this work, we propose a simple and controllable method for the synthesis of KFeS_2_ with diatomite morphology using a multistep sacrificial template.

## 2. Materials and Methods

All chemicals were purchased from Aladdin (Shanghai, China) and were of analytical purity and thus used without further purification. The diatomite material was also supplied from Aladdin.

### 2.1. Synthesis of FeOOH@D Nanorods on Diatomite (D)

MnO_2_@D was prepared by modifying a one-step hydrothermal method that has been previously published by our research group [6]. KMnO_4_ solution (70 mL, 0.05 M) and diatomite (100 mg) were placed into a Teflon-lined stainless-steel autoclave (Henan Gongyi Yuhua Instrument Co. LTD, Henan, China) that was subsequently maintained at 160 °C for 24 h. The sample was then removed, washed with distilled water and ethanol, and dried at 60 °C to obtain MnO_2_ composites. Subsequently, 150 mg FeSO_4_·7H_2_O and 80 mg MnO_2_@D were dissolved and dispersed in 70 mL of an ethylene glycol–water solution (ethylene glycol:water = 1:7) successively, and the mixture was transferred to a Teflon-lined stainless-steel autoclave and rotated at 120 °C for 12 h. After cooling to room temperature, the product was washed with de-ionized (DI) water and dried at 60 °C for 12 h.

### 2.2. Synthesis of Diatomite (D)-like KFeS_2_

The as-prepared FeOOH@D (100 mg), Na_2_S·9H_2_O (2.88 g), and KOH (19.6 g) were next dispersed and dissolved in 70 mL of the ethanol–water solution (water: ethanol = 1:1). Then, the mixture was transferred into a Teflon-lined stainless-steel autoclave and rotated constantly at 120 °C for 12 h. After cooling to room temperature, the product was washed with DI water and dried at 60 °C for 12 h.

### 2.3. Synthesis of FeS_2_-Modified Diatomite 

To create the FeS_2_-modified diatomite, the as-prepared FeOOH@D (100 mg) and Na_2_S·9H_2_O (2.88 g) were dispersed and dissolved in 70 mL of the ethanol–water solution (water:ethanol = 1:1). Then, the mixture was transferred into a Teflon-lined stainless-steel autoclave and constantly rotated at 120 °C for 12 h. After cooling to room temperature, this product was washed with DI water and dried at 60 °C for 12 h.

### 2.4. Synthesis of KFeS_2_

The MnO_2_ composites were prepared using a one-step hydrothermal method. As is typical, KMnO_4_ solution (70 mL, 0.05 M) was placed into a Teflon-lined stainless-steel autoclave at 160 °C for 24 h. The sample was then removed, washed with distilled water and ethanol, and dried at 60 °C to obtain the MnO_2_ composites.

Next, 150 mg FeSO_4_·7H_2_O and 80 mg MnO_2_ were then and dispersed in 70 mL of an ethylene glycol–water solution (ethylene glycol:water = 1:7) successively. The mixture was subsequently transferred to a Teflon-lined stainless-steel autoclave and rotated constantly at 120 °C for 12 h. After cooling to room temperature, the product was washed with DI water and dried at 60 °C for 12 h.

Finally, the as-prepared FeOOH (100 mg), Na_2_S·9H_2_O (2.88 g), and KOH (19.6 g) were dispersed and dissolved in 70 mL of the ethanol–water solution (water:ethanol = 1:1), and the mixture was then transferred into a Teflon-lined stainless-steel autoclave and rotated constantly at 120 °C for 12 h. After cooling to room temperature, the product was washed with DI water and dried at 60 °C for 12 h.

### 2.5. Characterization of Materials

The crystal structures of the KFeS_2_ and diatomite-like KFeS_2_ were determined by X-ray diffraction (XRD) (Panaco, Almelo, The Netherlands) at a scanning angle of 2θ = 5–80 degrees. The chemical constituents of the samples were determined by X-ray photoelectron spectroscopy (XPS) (Thermo Fisher Technology, Waltham, MA, USA) as well, and the micromorphology and structure were characterized using scanning electron microscopy (SEM) (Zeiss, Germany) at 5 kV and transmission electron microscopy (TEM) (Zeiss, Germany).

### 2.6. Electrochemical Measurements

A working electrode was prepared by mixing 70 wt% active materials (diatomite-like KFeS_2_), 20 wt% acetylene black, and 10 wt% polyvinylidene fluoride (PVDF) in N-methyl-2-pyrrolidone (NMP), and the slurry was spread onto a foam nickel current collector (1 × 1 cm^2^). This electrode was then heated to 120 °C for 12 h in order to evaporate the solvent and was then uniaxially pressed under 10 MPa. A three-electrode was then used to evaluate the capacitive performance of the electrode materials.

The electrochemical performance of the as-prepared electrode was carried out using the CHI 660E electrochemical station (Shanghai Chenhua Instrument Co., LTD, Shanghai, China). For the three-electrode configuration, the working electrodes (1 × 1 cm^2^) were the diatomite-like KFeS_2_, a platinum plate was used as the counter electrode, and silver chloride electrode was used as the reference electrode. Cyclic voltammetry (CV), linear sweep voltammetry (LSV), and galvanostatic charging/discharging (GCD) were employed to investigate the electrochemical performance of the composites, where the applied potential window ranged from 0 to 0.6 V in a 6 M KOH electrolyte. CVs were recorded at scan rates of 10, 20, 40, 50, 80, and 100 mV s^−1^, and GCD curves were obtained at constant current densities of 1, 2, 4, 5, 8, and 10 A g^−1^. CV and GCD had potential windows of 0.6 V and 0.45 V, respectively. Finally, electrochemical impedance spectroscopy (EIS) was conducted in the frequency range of 100 kHz to 0.01 Hz with a perturbation amplitude of 5 mV versus the open-circuit potential.

## 3. Results and Discussion

**Scheme 1:**
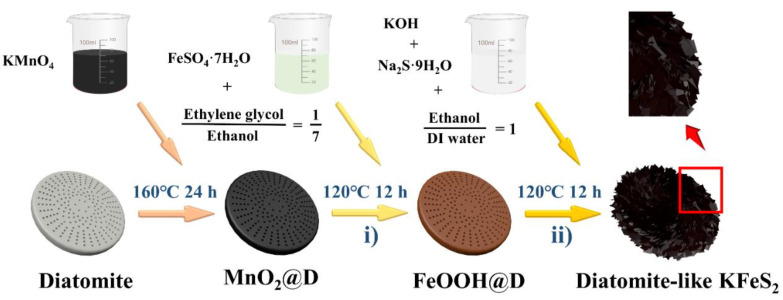
Multistep sacrificial template method to prepare biomimetic diatomite structured KFeS_2_ electrode materials with bifunction of supercapacitance and hydrolysis: (i) Conversion of MnO_2_ nanosheets to FeOOH nanorods on the surface of diatomite template; (ii) Etching of diatomite in FeOOH@D template and sulfation of FeOOH by KOH and Na_2_S·9H_2_0 at the same time to prepare diatomite-like KFeS_2_.

As shown in Scheme 1, the diatomite-like KFeS_2_ was synthesized using a sacrificial template method that combined the etching of sulfide and diatomite into one step. In the first step of the process, MnO_2_@diatomite (MnO_2_@D) was prepared using a hydrothermal method according to the self-decomposition of KMnO_4_. Then, the prepared MnO_2_ was replaced by hydroxyl iron oxide (FeOOH) based on the spontaneous redox reaction between Fe^2+^ and MnO_2_. After becoming vulcanized by Na_2_S·9H_2_O in the alcohol–water solution under high temperature, FeOOH was transformed into KFeS_2_ and the diatomite was dissolved by KOH, resulting in a hollow diatomite-like KFeS_2_ structure.

**Figure 1:**
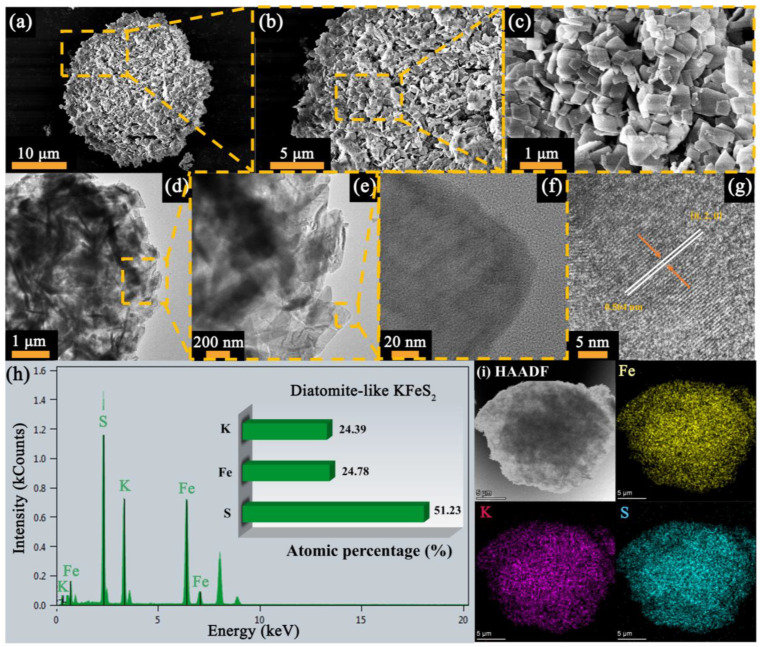
(**a**–**c**) Magnification increases for the SEM images of the diatomite-like KFeS_2_; (**d**–**f**) Magnification increases for the TEM images of the diatomite-like KFeS_2_; (**g**) Typical TEM image of the diatomite-like KFeS_2_ single wire; (**h**) EDS mapping of diatomite-like KFeS_2_; (**i**) HAADF-STEM images and corresponding EDS mapping of diatomite-like KFeS_2_.

Figure 1 shows the morphology of diatomite-like KFeS_2_. In Figure 1a, after the multistep sacrificial template method, the KFeS_2_ also kept a diatomite-like round cake shape that consisted of numerous nanosheets that joined together with each other to form a porous structure (Figure 1b,c). This structure could also be observed inform the TEM images (Figure 1d–f). This unique nanostructure definitely increased the SSA of KFeS_2_, which is very important for improving its electrochemical and OER properties. Moreover, a lattice spacing of 0.564 nm, as shown in Figure 1g, can be indexed to the (0 2 0) plane of KFeS_2_, and the typical EDS spectrum in Figure 1h reveals that the atomic percentage of elements K, Fe, and S followed a ratio of 1:1:2, which indicates the presence of KFeS_2_. Furthermore, the element mapping images and scatter superimposed graph in Figure 1i also show that K, Fe, and S were uniformly distributed in the material. These results point to the successful synthesis of KFeS_2_ with the 3D morphology and dimensions of diatomite.

**Figure 2:**
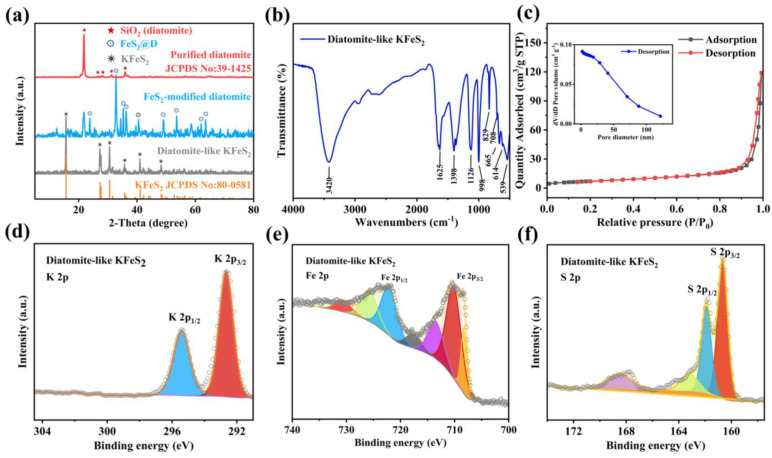
(**a**) XRD patterns of purified diatomite, KFeS_2_ with diatomite morphology, and FeS_2_-modified diatomite; (**b**) FT-IR spectra of diatomite-like KFeS_2_ in the range of 500–4000 cm^−1^; (**c**) N_2_ adsorption–desorption isotherms and the corresponding pore size distribution of the diatomite-like KFeS_2_; high-resolution XPS spectra of K 2p, Fe 2p, and S 2p of the diatomite-like KFeS_2_ (**d**–**f**), respectively.

For comparison, FeS_2_-modified diatomite was produced but without diatomite etching. Figure 2a shows the composition and crystalline phase of purified diatomite, FeS_2_-modified diatomite, and diatomite-like KFeS_2_. The diffraction peaks of purified diatomite were observed at 22.0°, 28.4°, 31.5°, and 36.1°, which refer to SiO_2_ (JCPDS No. 39-1425, α = 4.937 Å, b = 4.937 Å, c = 6.924 Å), indicating a chemical composition of diatomite. For FeS_2_-modified diatomite, only weak diffraction peaks were found for diatomite because the template of diatomite was not etched. However, the diffraction peaks of diatomite-like KFeS_2_ were observed as 15.6°, 27.4°, 30.7°, and 40.9° (JCPDS No. 80-0581, α = 7.089 Å, b = 11.304 Å, c = 5.398 Å), demonstrating its high crystallinity, and the average crystallites size is about 27.5 nm. Additionally, the diffraction peaks of purified diatomite could not be observed, which confirmed that the diatomite was completely etched by KOH. Moreover, the sample without etching was also studied and clearly showed that the synthesis of KFeS_2_ had occurred as a product of the co-heating of KOH with Na_2_S·9H_2_O and hydroxy-iron oxide. Figure 2b depicts the FTIR spectrum of the diatomite-like KFeS_2_. The peak at 3420 cm^−1^ corresponds to strong stretching vibrations of the O–H bond (H_2_O), and the bands at 1126 cm^−1^ and 829 cm^−1^ correspond to the asymmetric stretching of sulfur functional groups (S–O). Finally, the peaks at 708 cm^−1^, 665 cm^−1^, 614 cm^−1^, and 539 cm^−1^ correspond to disulfide stretching vibrations (S–S) [34].

The surface properties of the prepared diatomite-like KFeS_2_ were tested by Brunauer–Emmett–Teller (BET) and N_2_ adsorption–desorption measurements. Figure 2c shows the corresponding N_2_ adsorption–desorption isotherms and the pore size distribution curves of the samples. As shown in Figure 2c, the isotherm surface is that of a typical mesoporous material with a sharp increase in N_2_ adsorption near a relative pressure of 1, which indicates the presence of macropores in the diatomite-like KFeS_2_. This sharp increase is due to the presence of macropores on the surface of the diatomite template. According to BET analysis, the synthesized diatomite-like KFeS_2_ exhibited a large specific surface area of 42.35 m^2^ g^−1^, which is much higher than that of the modified diatomite studied in our previous work [35]. From the pore size distribution curves, the diatomite-like KFeS_2_ shows a wide pore size spread, and the average pore diameter was 29.03 nm in 4 V A^−1^ by BET, which formed a layered porous structure possessed by the composite. This type of layered surface morphology with good pore structure is beneficial for enhancing the performance of electrochemical capacitors because the large pore channels allow fast electrolyte transport, and the small pores provide more active sites for chemical reactions to take place.

To explore the chemical composition of the structure further, X-ray photoelectron spectroscopy (XPS) was performed to examine the elemental states of the diatomite-like KFeS_2_. The corresponding high-resolution spectra of K, Fe, and S are displayed in Figure 2d–f. The XPS spectrum of K 2p in Figure 2d shows two typical peaks at 295.40 eV and 292.61 eV, corresponding to the K 2p_1/2_ and K 2p_3/2_ spin–orbit peaks of diatomite-like KFeS_2_, respectively. Figure 2e shows the spectra of Fe 2p for the diatomite-like KFeS_2_, where the peaks located at binding energies 722.53 eV and 708.32 eV belong to Fe 2p_1/2_ and Fe 2p_3/2_, respectively, suggesting the presence of Fe^3+^ [36,37]. In Figure 2f, the peaks at 161.81 eV and 160.67 eV correspond to S 2p_1/2_ and S 2p_3/2_, respectively, and we attribute the additional weak peak at 167.74 eV to a sulfur–oxygen bond that was possibly caused by air contact [38].

**Figure 3:**
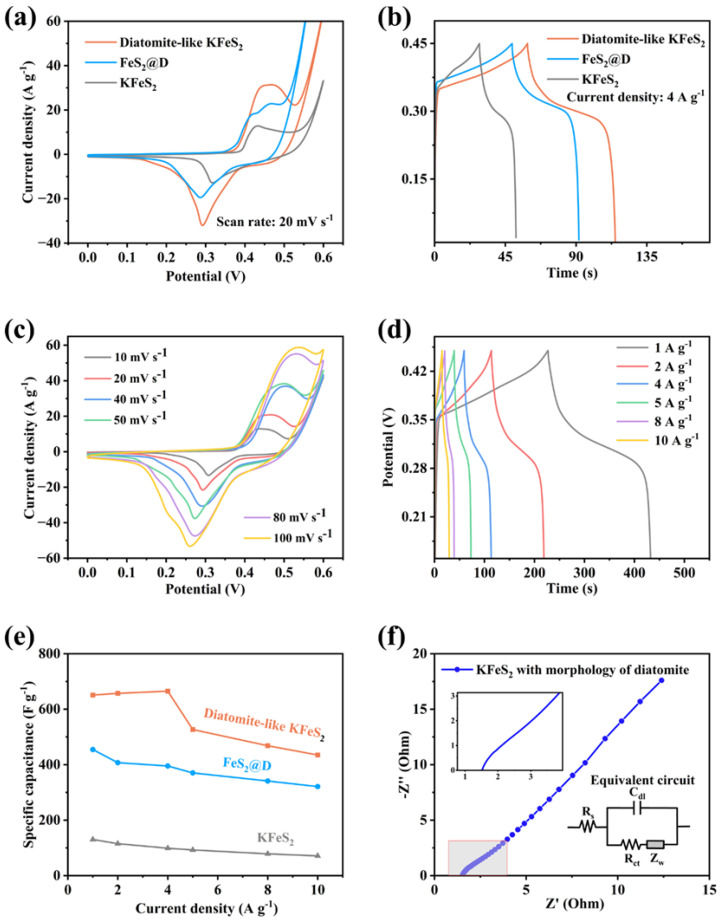
(**a**) The comparison of cyclic voltammograms at a scan rate of 20 mV s^−1^ between diatomite-like KFeS_2_, FeS_2_@D, and KFeS_2_; (**b**) GCD curves of diatomite-like KFeS_2_, FeS_2_@D, and KFeS_2_ at a current density of 4 A g^−1^; (**c**) cyclic voltammograms of diatomite-like KFeS_2_ composite at different scan rate (10, 20, 40, 50, 80, and 100 mV s^−1^) in a 6 M KOH aqueous electrolyte; (**d**) GCD curves of diatomite-like KFeS_2_ electrodes at different current densities (1, 2, 4, 5, 8, and 10 mA cm^−2^); (**e**) specific capacitances of diatomite-like KFeS_2_, FeS_2_@D, and KFeS_2_ measured under different current densities; (**f**) the electrochemical impedance spectrum of the diatomite-like KFeS_2_ electrodes at open circuit potential in the frequency range from 0.01 Hz to 100 kHz (the inset is a partial enlargement of the curve).

Next, in order to compare the electrochemical performance of diatomite-like KFeS_2_, FeS_2_@D, and KFeS_2_, the CV curves at a scan rate of 20 mV s^−1^ with GCD curves at a current density of 4 A g^−1^ are shown in Figure 3a,b. Evidently, the area surrounded by the curve of the diatomite-like KFeS_2_ was larger than the others. The GCD curves of the diatomite-like KFeS_2_, FeS_2_@D, and KFeS_2_ are shown in Figure 3b. The discharge time of the diatomite-like KFeS_2_ was longer than that of FeS_2_@D or KFeS_2_, which indicates its larger capacitance. In addition, the areas of the CV curves of the diatomite-like KFeS_2_ and FeS_2_@D were larger than that of KFeS_2_, and their discharge times were also longer than KFeS_2_, indicating enhancement in electrochemical capacitance from diatomite morphology. Additionally, the CV curves of the diatomite-like KFeS_2_ electrodes in 6 M KOH aqueous electrolyte at various scan rates are shown in Figure 3c, where an obvious redox peak can be seen that indicates that a typical faradaic pseudo reaction occurred between the KFeS_2_ electrode and the KOH electrolyte. As shown in Figure 3d, stable platforms can also be observed in the charge–discharge curves, indicating a pseudo-reaction response of the active material. The specific capacitance C_m_ (F g^−1^) can be calculated using Equation (1):

(1) $$C_{m}=\frac{\int^{}\mathrm{Udt}\times I}{0.5m\times \Delta V^{2}}$$

where I is the discharging current, U is the potential, t is the discharging time, and m is the weight of the active materials. The specific capacitance of diatomite-like KFeS_2_ was calculated to be 651 F g^−1^ at a current density of 1.0 A g^−1^, highlighting its unique porous structure and high SSA. High porosity makes it easier for ions to be transferred into the structure, leading to more redox reactions and surface adsorption of electrolyte ions. 

Table 1 shows the comparison of iron-based sulfide electrodes and highlights the excellent electrochemical performance of diatomite-like KFeS_2_. In addition, the rate capabilities of diatomite-like KFeS_2_, FeS_2_@D, and KFeS_2_ are displayed in Figure 3e. Obviously, the slope of the diatomite-like KFeS_2_ was lower than that of FeS_2_@D or KFeS_2_, meaning that the diatomite-like KFeS_2_ had a better rate capability. The overall specific capacitance of the diatomite-like KFeS_2_ also showed a slightly decreasing trend as current density increased from 1.0 to 10.0 A g^−1^. However, a slight increase at low current density (1.0 to 4.0 A g^−1^) can be ascribed to the thermal effect caused by repeated charging and discharging of the electrode [39]. After increasing the current density to 10 A g^−1^, about 66.8% of the initial capacitance remained for the diatomite-like KFeS_2_ electrode, which serves to highlight is rate capability further. Specifically, this rate in FeS_2_@D was about 70.6% and in KFeS_2_ was about 54.6%. 

The rate capabilities of the diatomite-like KFeS_2_ and FeS_2_@D were both better than KFeS_2_, which indicates improvement from introducing the diatomite morphology. Furthermore, Figure S1 shows the cycle performance of the diatomite-like KFeS_2_ at a current density of 4 A g^−1^. Here, we see that the impedance of the diatomite-like KFeS_2_ electrode was calculated over a frequency range of 100 kHz–0.01 Hz by applying an AC voltage with an amplitude of 5 mV at the open circuit potential. As shown in Figure 3f, the impedance patterns had a half arc at high frequencies and a linear part at low frequencies. The equivalent circuit of the Nyquist plot is shown in Figure 3f, where we can see that the impedance arc in the high frequencies region can be replaced by an interfacial Faraday charge transfer resistor (R_ct_) and a parallel constant phase element (C_d_) for the double layer capacitance. A straight line with a slope of 45° in the mid-frequency region along the arc indicates the finite-length diffusive Warburg impedance (Z_w_), which is associated with the diffusion/transport of electrolyte ions in the electrode. The vertical line in the very low frequency region shows the ideal capacitive behavior of the diatomite-like KFeS_2_ electrode material, and the EIS results show the pseudo-capacitive properties and porous structure characteristics of the diatomite-like KFeS_2_ electrode.

**Figure 4:**
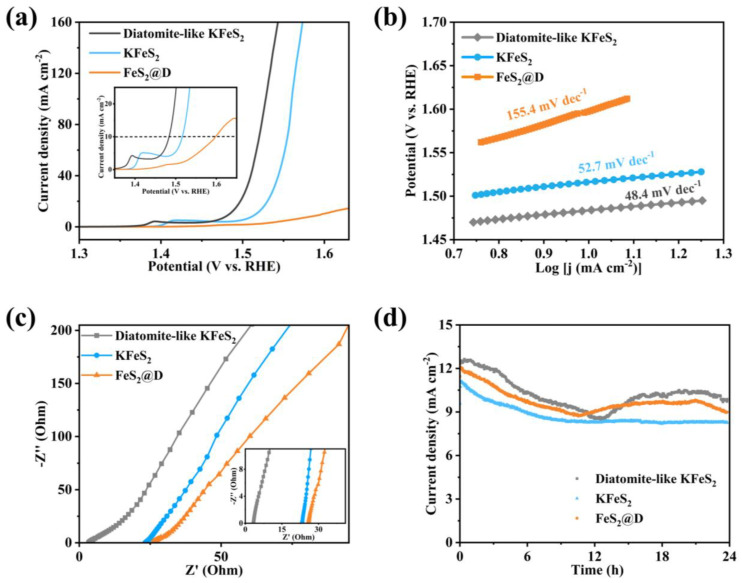
(**a**) LSV plots (the inset is the partial enlargement plot); (**b**) the corresponding Tafel plots; (**c**) comparison of EIS between diatomite-like KFeS_2_, KfeS_2_, and FeS_2_@D (the partial enlargement plot is shown in the inset); (**d**) current–time curves of diatomite-like KFeS_2_, KFeS_2_, and FeS_2_@D for 24 h.

The catalytic performance of the diatomite-like KFeS_2_ was also evaluated in 1 M KOH. For comparison, FeS_2_-modified diatomite and iron-based sulfide without diatomite morphology (KFeS_2_) were set as controls. The electrocatalytic activities of the diatomite-like KFeS_2_, FeS_2_@D, and KFeS_2_ nanostructured electrodes were investigated using linear sweep voltammetry (LSV) measurements at a scan rate of 5.0 mA s^−1^, and the results are shown in Figure 4a. The overpotential (ƞ) for the diatomite-like KFeS_2_ was estimated to be ~0.25 mV, which was much lower than both KFeS_2_ (~0.29 mV) and FeS_2_@D (~0.37 mV at a current density of 10 mA cm^−2^. In addition, the Tafel slopes of the diatomite-like KFeS_2_, KFeS_2_, and FeS_2_@D were estimated as 48.4 mV dec^−1^, 52.7 mV dec^−1^, and 155.4 mV dec^−1^, respectively, as shown in Figure 4b. The Tafel slope of the diatomite-like KFeS_2_ was lower than the other two structures, indicating its superior reaction kinetics characteristics and excellent oxygen evolution performance. The EIS impedance spectrum in Figure 4c also shows that the impedance of the diatomite-like KFeS_2_ was much lower than that of KFeS_2_ or FeS_2_@D, which indicates its superior electrical conductivity. The lowest impedance of diatomite-like KFeS_2_ favors the transfer of electrons and results in the lowest overpotential among the three substances as well. This low impedance is also beneficial for the adsorption and desorption of intermediates during the reaction process and contributes to the excellent catalytic performance of diatomite-like KFeS_2_.

Cycling performance was further tested using the time–current method, and the results for the diatomite-like KFeS_2_, KFeS_2_, and FeS_2_@D at an initial potential of 0.53 V, 0.62 V, and 0.67 V for each after 24 h are shown in Figure 4d. Analysis of the time–current curve results shows that the cycling stability of the diatomite-like KFeS_2_ and FeS_2_@D were much higher than that of the KFeS_2_ without the morphology of diatomite. The cyclic curves of the diatomite-like KFeS_2_ and FeS_2_@D were noticeably similar, indicating that the morphology of diatomite made a central contribution to its cycling performance.

The electrocatalytic performance of catalysts for hydrolysis is highly dependent on their catalytic activity and the number of active sites, which are closely related to the morphology, size, and structure of the catalyst [45]. Indeed, we found that the extremely low overpotential, good electrical conductivity, and superior cycling stability of the diatomite-like KFeS_2_ synthesized in this study were due to its unique diatomite nanostructure, which increased the number of active centers. According to the adsorbate evolution mechanism (AEM), an increased number of metal active sites can adsorb more intermediates and can facilitate the synergistic effect of Fe and S, thus accelerating the electrocatalytic performance of water decomposition [46]. Finally, we provide a table of comparisons (see Table 2) of the results for the diatomite-like KFes_2_ with other sulfides that have been reported earlier, and these results show that the prepared diatomite-like KFeS_2_ had excellent OER performance.

## 4. Conclusions

In summary, diatomite-like KFeS_2_ was successfully synthesized using a simple multistep sacrificial template method. The nanostructure exhibited the potential for a broad set of applications, such as in supercapacitor anodes and electrocatalysts for OERs. The diatomite-like KFeS_2_ also exhibited extremely high specific capability (651 F g^−1^ at 1.0 A g^−1^). In addition, as an electrocatalyst, the diatomite-like KFeS_2_ possessed a lower overpotential (ƞ_10_ of 254 mV at a current density of 10 mA cm^−2^) during OERs, compared to other iron-based sulfide composite materials. These results indicate that diatomite-like KFeS_2_ shows promise for use in the assembly of supercapacitors and oxygen precipitation reactions.

## Figures and Tables

**Table 1:** Various iron-based sulfide electrode materials with their specific capacitance.

No.	Materials	Morphology	Electrolyte	Specific Capacitance	Reference
1	Diatomite-like KFeS_2_	Diatomite-like	6 M KOH	651 F g^−1^ at 1 A g^−1^	This work
2	FeS_2_ CNFs	Nanosphere	6 M KOH	203.4 F g^−1^ at 1 A g^−1^	[20]
3	Fruit-like FeS_2_@Carbon microspheres	Microsphere	1 M KOH	278.4 F g^−1^ at 1 A g^−1^	[40]
4	N-doped FeS_2_ nanosphere	Nanosphere	0.5 M NaOH	238.2 mF g^−1^ at 3 mA g^−1^	[41]
5	50% FeS_2_/3DPC (FeS_2_ content at 50% weight percent)	Nanoparticles	1 M KOH	304 F g^−1^ at 2 A g^−1^	[30]
6	Donator-FeS/C	Nanoparticles	2 M KOH	275.65 F g^−1^ at 30 mA cm^−2^	[42]
7	FeS_2_/PVP/NF	Nanoparticles	3 M KOH	526.08 F g^−1^ at 1 A g^−1^	[12]
8	FeS_x_ grown on stainless steel	Cuboidal-like	1 M Na_2_SO_4_	730 mF g^−1^ at 1 mA g^−1^	[43]
9	Hierarchical FeS/RGO/FeS@Fe foil	Nanosheets	2 M KOH	206.25 F g^−1^ at 20 mA cm^−2^	[44]

**Table 2:** Various iron-based sulfide electrode materials with their Tafel slopes and overpotentials.

No.	Materials	Morphology	Tafel Slope (mV dec^−1^)	Overpotential (mV)	Reference
1	Diatomite-like KFeS_2_	Diatomite-like	48.4	254@10 mA cm^−2^	This work
2	FeNi(OH)_x_/FeS/IF	Nanosheets	53	273@10 mA cm^−2^	[47]
3	FeS/Fe_2_O_3_ heterogeneous nanosheets	Nanosheets	51.71	266.5@10 mA cm^−2^	[16]
4	Fe_2_O_3_/FeS	Nanorods	90	370@40 mA cm^−2^	[15]
5	Hybrid nanoarray	Nanoarray	80	260@10 mA cm^−2^	[14]
6	FeS-Co_9_S_8_/IF	Heterostructure	50.3	332@500 mA cm^−2^	[48]
7	FeS	-	69	320@10 mA cm^−2^	[49]
8	FeS_x_/CF	Heterostructure	105	340@10 mA cm^−2^	[50]
9	FeS_2_@MXene	Nanoparticles	58.6	240@10 mA cm^−2^	[51]

## Data Availability

Data of the compounds are available from the authors.

## Supplementary Materials

The following supporting information can be downloaded at: https://www.mdpi.com/article/10.3390/nano13040643/s1, Figure S1: Cycle performance of diatomite-like KFeS_2_.

## References

[1] Kim J., Lee J., Liu C., Pandey S., Joo S.W., Son N., Kang M. (2021). Achieving a long-term stability by self-redox property between Fe and Mn ions in the iron-manganese spinel structured electrode in oxygen evolution reaction. Appl. Surf. Sci..

[2] Liu J.H., He X., Wang Y.Y., Sun Z.J., Liu Y.S., Liu B.S., Li H.D., Guo F., Li X. (2022). Deep reconstruction of highly disordered iron/nickel nitrate hydroxide nanoplates for high-performance oxygen evolution reaction in alkaline media. J. Alloy. Compd..

[3] Zhang G.G., Yuan J.Y., Liu Y., Lu W., Fu N.Q., Li W.F., Huang H.T. (2018). Boosting the oxygen evolution reaction in non-precious catalysts by structural and electronic engineering. J. Mater. Chem. A.

[4] Wang C.H., Li C.M., Liu J.L., Guo C.X. (2021). Engineering transition metal-based nanomaterials for high-performance electrocatalysis. Mater. Rep. Energy.

[5] Chen W., Rakhi R.B., Hu L.B., Xie X., Cui Y., Alshareef H.N. (2011). High-Performance Nanostructured Supercapacitors on a Sponge. Nano Lett..

[6] Zhang Y.X., Huang M., Li F., Wang X.L., Wen Z.Q. (2014). One-pot synthesis of hierarchical MnO_2_-modified diatomites for electrochemical capacitor electrodes. J. Power Sources.

[7] Simon P., Gogotsi Y. (2008). Materials for electrochemical capacitors. Nat. Mater..

[8] Zeng Y., Yu M., Meng Y., Fang P., Lu X., Tong Y. (2016). Iron-Based Supercapacitor Electrodes: Advances and Challenges. Adv. Energy Mater..

[9] Owusu K.A., Qu L., Li J., Wang Z., Zhao K., Yang C., Hercule K.M., Lin C., Shi C., Wei Q. (2017). Low-crystalline iron oxide hydroxide nanoparticle anode for high-performance supercapacitors. Nat. Commun..

[10] Bu R.R., Deng Y., Wang Y.L., Zhao Y., Shi Q.Q., Zhang Q., Xiao Z.Y., Li Y.Y., Sun W., Wang L. (2021). Bucket Effect: A Metal-Organic Framework Derived High-Performance FeS_2_/Fe_2_O_3_@S-rGO Negative Material for Enhanced Overall Supercapacitor Capacitance. ACS Appl. Energ. Mater..

[11] Chen X.L., Shi T., Zhong K.L., Wu G.L., Lu Y. (2020). Capacitive behavior of MoS_2_ decorated with FeS_2_@carbon nanospheres. Chem. Eng. J..

[12] Durga I.K., Rao S.S., Kalla R.M.N., Ahn J.W., Kim H.J. (2020). Facile synthesis of FeS_2_/PVP composite as high-performance electrodes for supercapacitors. J. Energy Storage.

[13] Pham D.T., Baboo J.P., Song J., Kim S., Mathew V., Alfaruqi M.H., Sambandam B., Kim J. (2018). Facile synthesis of pyrite (FeS_2_/C) nanoparticles as an electrode material for non-aqueous hybrid electrochemical capacitors. Nanoscale.

[14] Luan X.Q., Du H.T., Kong Y., Qu F.L., Lu L.M. (2019). A novel FeS-NiS hybrid nanoarray: An efficient and durable electrocatalyst for alkaline water oxidation. Chem. Commun..

[15] Khan N.A., Rashid N., Ahmad I., Zahidullah, Zairov R., Rehman H.U., Nazar M.F., Jabeen U. (2022). An efficient Fe_2_O_3_/FeS heterostructures water oxidation catalyst. Int. J. Hydrog. Energy.

[16] Guo W.H., Zhang Q., Fu H.C., Yang Y.X., Chen X.H., Luo H.Q., Li N.B. (2022). Semi-sacrificial template growth of FeS/Fe_2_O_3_ heterogeneous nanosheets on iron foam for efficient oxygen evolution reaction. J. Alloy. Compd..

[17] Xue Y.R., Liu M.Y., Qin Y.Y.X., Zhang Y.F., Zhang X.J., Fang J.J., Zhang X., Zhu W., Zhuang Z.B. (2022). Ultrathin NiFeS nanosheets as highly active electrocatalysts for oxygen evolution reaction. Chin. Chem. Lett..

[18] Feng D., Zhang X., Sun Y., Ma T. (2020). Surface-defective FeS_2_ for electrochemical NH_3_ production under ambient conditions. Nano Mater. Sci..

[19] Huang F., Wu R., Li J., Sun Y.F., Jian J.K. (2013). Synthesis of NaFeS_2_ Nanorods by Salvothermal Technique, International Forum on Mechanical and Material Engineering (IFMME 2013).

[20] Huang Y., Zhao H., Bao S., Yin Y., Zhang Y., Lu J. (2022). Hollow FeS_2_ nanospheres encapsulated in N/S co-doped carbon nanofibers as electrode material for electrochemical energy storage. J. Alloy. Compd..

[21] Li L.X., Gao T.Y., Ge Y.S., Zhang Q., Wang J.Y., Ma Z.P., Guo W.F., Yu S.X., Fan Y.Q. (2021). Ultra-long KFeS_2_ nanowires grown on Fe foam as a high-performance anode for aqueous solid-state energy storage. J. Mater. Chem. A.

[22] Liang D.X., Ji M.H., Zhu S.Y., Chen Y., Wang Z.H., Liu Y.W., Khan A.H.R., Ri Y.H., Yu H.B., Huo M.X. (2021). A novel Fe recycling method from pickling wastewater producing a KFeS_2_ whisker for electroplating wastewater treatment. Environ. Sci. Wat. Res. Technol..

[23] Tufa L.T., Gicha B.B., Wu H., Lee J. (2021). Fe-Based Mesoporous Nanostructures for Electrochemical Conversion and Storage of Energy. Batter. Supercaps.

[24] Yang J., Chen J.W., Wang Z.X., Wang Z., Zhang Q.C., He B., Zhang T., Gong W.B., Chen M.X., Qi M. (2021). High-Capacity Iron-Based Anodes for Aqueous Secondary Nickel-Iron Batteries: Recent Progress and Prospects. Chem. Electro. Chem..

[25] Fang Y.J., Chen Z.X., Xiao L.F., Ai X.P., Cao Y.L., Yang H.X. (2018). Recent Progress in Iron-Based Electrode Materials for Grid-Scale Sodium-Ion Batteries. Small.

[26] Li B., Pang H., Xue H.G. (2021). Fe-based phosphate nanostructures for supercapacitors. Chin. Chem. Lett..

[27] Wu X.Y., Markir A., Xu Y.K., Zhang C., Leonard D.P., Shin W., Ji X.L. (2019). A Rechargeable Battery with an Iron Metal Anode. Adv. Funct. Mater..

[28] Jayathilake B.S., Plichta E.J., Hendrickson M.A., Narayanan S.R. (2018). Improvements to the Coulombic Efficiency of the Iron Electrode for an All-Iron Redox-Flow Battery. J. Electrochem. Soc..

[29] Liu W.W., Zhu M.H., Liu J.H., Li X., Liu J. (2019). Flexible asymmetric supercapacitor with high energy density based on optimized MnO_2_ cathode and Fe_2_O_3_ anode. Chin. Chem. Lett..

[30] Wu Z.-S., Sun Y., Tan Y.-Z., Yang S., Feng X., Muellen K. (2012). Three-Dimensional Graphene-Based Macro- and Mesoporous Frameworks for High-Performance Electrochemical Capacitive Energy Storage. J. Am. Chem. Soc..

[31] Yu Y., Addai-Mensah J., Losic D. (2010). Synthesis of Self-Supporting Gold Microstructures with Three-Dimensional Morphologies by Direct Replication of Diatom Templates. Langmuir.

[32] Dai X., Rao J., Bao Z., Li K., Feng L., Song D., Zhao L., Li W., Liu X., Yi S. (2022). Magnetic double-core@shell MnO_2_@NiFe@DE as a multifunctional scavenger for efficient removal of tetracycline, anionic and cationic dyes. J. Colloid Interface Sci..

[33] Li K., Teng H., Sun Q., Li Y., Wu X., Dai X., Wang Y., Wang S., Zhang Y., Yao K. (2022). Engineering active sites on nitrogen-doped carbon nanotubes/cobaltosic oxide heterostructure embedded in biotemplate for high-performance supercapacitors. J. Energy Storage.

[34] Philias J.M., Marsan B. (1999). FTIR spectroscopic study and thermal and electrical properties of polymer electrolytes containing a cesium thiolate/disulfide redox couple. Electrochim. Acta.

[35] Sprynskyy M., Gadzala-Kopciuch R., Nowak K., Buszewski B. (2021). Removal of zearalenone toxin from synthetics gastric and body fluids using talc and diatomite: A batch kinetic study. Colloids Surf. B.

[36] Ni J.F., Sun M.L., Li L. (2019). Highly Efficient Sodium Storage in Iron Oxide Nanotube Arrays Enabled by Built-In Electric Field. Adv. Mater..

[37] Wang M.X., Wang L.S., Yue G.H., Wang X., Yan P.X., Peng D.L. (2009). Single crystal of CuFeS_2_ nanowires synthesized through solventothermal process. Mater. Chem. Phys..

[38] Yang J., Zhang Q.C., Wang Z.X., Wang Z., Kang L.X., Qi M., Chen M.X., Liu W., Gong W.B., Lu W.B. (2020). Rational Construction of Self-Standing Sulfur-Doped Fe_2_O_3_ Anodes with Promoted Energy Storage Capability for Wearable Aqueous Rechargeable NiCo-Fe Batteries. Adv. Energy Mater..

[39] Wang K., Wang J., Wu Y., Zhao S., Wang Z., Wang S. (2016). Millisecond photo-thermal process on significant improvement of supercapacitor’s performance. Appl. Therm. Eng..

[40] Liu X.X., Deng W.Z., Liu L.M., Wang Y.F., Huang C.F., Wang Z.J. (2022). Passion fruit-like microspheres of FeS_2_ wrapped with carbon as an excellent fast charging material for supercapacitors. New J. Chem..

[41] Wang Q., Liu Q.Q., Ni Y.L., Yang Y., Zhu X.F., Song Y. (2021). N-Doped FeS_2_ Achieved by Thermal Annealing of Anodized Fe in Ammonia and Sulfur Atmosphere: Applications for Supercapacitors. J. Electrochem. Soc..

[42] Cai J., Lai C.L., Sun P.X., Cui B.L., Yang C. (2022). 3 electrons donator-FeS/C composite anode for supercapacitor. J. Mater. Sci. Mater. Electron..

[43] Shao X.X., Zhu Z.Q., Zhao C.J., Zhao C.H., Qian X.Z. (2018). Hierarchical FeS/RGO/FeS@Fe foil as high-performance negative electrode for asymmetric supercapacitors. Inorg. Chem. Front..

[44] Upadhyay K.K., Nguyen T., Silva T.M., Carmezim M.J., Montemor M.F. (2019). Pseudocapacitive behaviour of FeS_x_ grown on stainless steel up to 1.8 V in aqueous electrolyte. J. Energy Storage.

[45] Zhang K., Zou R. (2021). Advanced Transition Metal-Based OER Electrocatalysts: Current Status, Opportunities, and Challenges. Small.

[46] Song J., Wei C., Huang Z.-F., Liu C., Zeng L., Wang X., Xu Z.J. (2020). A review on fundamentals for designing oxygen evolution electrocatalysts. Chem. Soc. Rev..

[47] Chen Q.W., Zhang X.Y., Dong Y.W., Guo B.Y., Ma Y., Wang L., Lv R.Q., Zhou Y.L., Chai Y.M., Dong B. (2020). Ultrafast surface modification of FeS nanosheet arrays with Fe-Ni bimetallic hydroxides for efficient oxygen evolution. J. Alloy. Compd..

[48] Liu H.J., Yu N., Yuan X.Q., Zhao H.Y., Zhang X.Y., Chai Y.M., Dong B. (2022). 0D-2D Schottky heterostructure coupling of FeS nanosheets and Co_9_S_8_ nanoparticles for long-term industrial-level water oxidation. Nano Res..

[49] Shankar A., Elakkiya R., Maduraiveeran G. (2020). Self-supported fabrication and electrochemical water splitting study of transition-metal sulphide nanostructured electrodes. New J. Chem..

[50] Wang W.B., Xu R.D., Yu B.H., Wang X.B., Feng S.Y. (2019). Electrochemical fabrication of FeS_x_ films with high catalytic activity for oxygen evolution. Rsc Adv..

[51] Xie Y.Y., Yu H.Z., Deng L.M., Amin R.S., Yu D.S., Fetohi A.E., Maximov M.Y., Li L.L., El-Khatib K.M., Peng S.J. (2022). Anchoring stable FeS_2_ nanoparticles on MXene nanosheets via interface engineering for efficient water splitting. Inorg. Chem. Front..

