# Supplementary material for: Diatomite-like KFeS2 for Use in High-Performance Electrodes for Energy Storage and Oxygen Evolution
Source: Nanomaterials (Basel). 2023 Feb 6;13(4):643. doi: 10.3390/nano13040643 (PMC9967514; doi:10.3390/nano13040643)
Supplement: Supplementary file 1 [file nanomaterials-13-00643-s001.zip › nanomaterials-2145645-supplementary.pdf]

Supporting Materials

Diatomite-Like $KFeS_2$ for Use in High-Performance Electrodes for Energy Storage and Oxygen Evolution

Can Wang [1], Kailin Li [1], Qing Sun [2], Shijin Zhu [1], Chenzhi Zhang [1], Yunhao Zhang [3], Zhongyi Shi [4], Youzhong Hu [1] and Yuxin Zhang [1,*]

1 College of Materials Science and Engineering, Chongqing University, Chongqing 400044, China
2 Multi-scale Porous Materials Center, School of Chemistry and Chemical Engineering, Chongqing University, Chongqing 400044, China
3 School of Energy and Power Engineering, Chongqing University, Chongqing 400044, China
4 Undergraduate School, Chongqing University, Chongqing 400044, China
* Correspondence: zhangyuxin@cqu.edu.cn

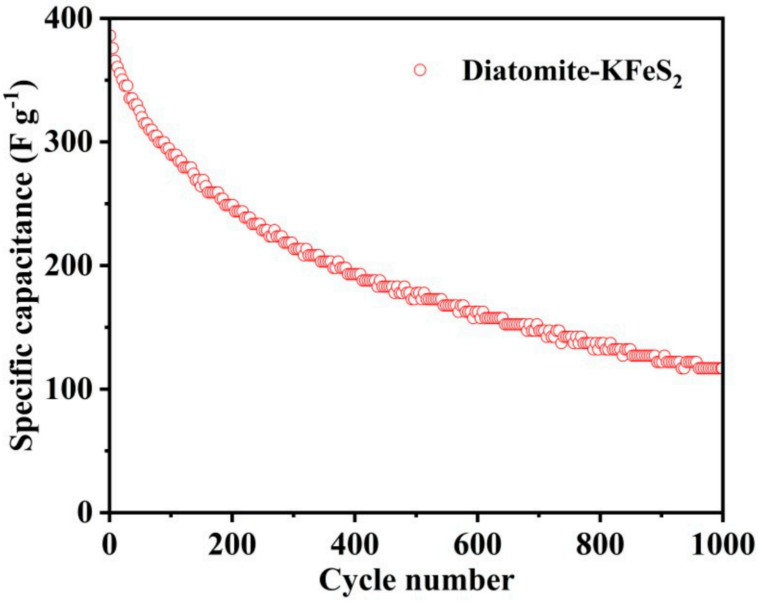

Figure S1. cycle performance of diatomite-like $KFeS_2$.
